# Cardiovascular risk factors among Roma and non-Roma populations in underprivileged settlements

**DOI:** 10.1186/s12875-024-02555-6

**Published:** 2024-08-08

**Authors:** Lilla Andréka, Orsolya Csenteri-Bárdos, Gergő József Szőllősi, Péter Andréka, Zoltán Jancsó, Péter Vajer

**Affiliations:** 1 Department of Family Medicine, Semmelweis University, Budapest, 1085 Hungary; 2 Doctoral College of Semmelweis University, Budapest, 1085 Hungary; 3 Gottsegen György National Cardiovascular Center, Budapest, 1096 Hungary; 4 Coordination Center for Research in Social Sciences, Faculty of Economics and Business, University of Debrecen, Debrecen, Hungary

**Keywords:** Health status, Cardiovascular risk factor, Roma, Non-Roma

## Abstract

**Background:**

The ‘Taking the screening tests close to the people’ program offers cardiovascular screening to the inhabitants of underprivileged settlements. This study aimed to evaluate the cardiovascular risk factors of underprivileged populations, including individuals who described themselves as belonging to the Roma population.

**Methods:**

During the program, we collected information about demographic features, lifestyle and current illnesses. A general health assessment (body weight, height, blood pressure and fasting blood glucose) and cardiovascular examination were performed. We analysed data on both Roma and non-Roma groups and used Pearson’s chi-squared test and multiple logistic regression models to analyse the factors that contribute to the onset of comorbidities, with a special focus on ethnicity.

**Results:**

Data from 6211 participants were processed. Based on self-reports, the non-Roma population consisted of 5352 respondents (1364 men (25.5%) and 3988 women (74.5%)), and the Roma population comprised 859 respondents (200 men (23.3%) and 659 women (76.7%)). A total of 91.2% (4849) of the non-Roma population and 92.5% (788) of the Roma population exercised less than 3 h per week (*p* < 0.001). Of the non-Roma population, 71.7% (3512) had a body mass index above 25 kg/m^2^, while the corresponding figure was 72.4% (609) in the Roma population (*p* = 0.709). The median body mass index was 28.0 (24.6–31.9) in the non-Roma population and 28.8 (24.5–33.0) in the Roma population (*p* < 0.001). The prevalence of active smokers was 28.7% (1531) in the non-Roma population and 60.3% (516) in the Roma population (*p* < 0.001). The prevalence of hypertension was 54.9% (2824) in the non-Roma population and 49.8% (412) in the Roma population (*p* < 0.001). The prevalence of diabetes was 11.5% (95) in the Roma population and 12.2% (619) in the non-Roma population (*p* < 0.001).

**Conclusion:**

We found a high prevalence of overweight and obesity, a lack of physical activity and an remarkably high smoking rate in the studied underprivileged population. Both type 2 diabetes and hypertension were more common among people living in underprivileged settlements than in the general Hungarian population. People living in underprivileged settlements need more attention in primary care.

**Supplementary Information:**

The online version contains supplementary material available at 10.1186/s12875-024-02555-6.

## Background

Cardiovascular diseases (CVD) are the leading cause of adult morbidity and mortality in most developed countries [1, 2], accounting for 46.8% of deaths in Hungary in 2022 [3]. The most important risk factors for CVD include gender, age, high blood pressure, high cholesterol level, diabetes, abdominal obesity, smoking, a sedentary lifestyle, a diet low in fruits and vegetables, psychosocial stress, and a family history of CVD [4].

Of the 13 national minorities in Hungary, the Roma population is the largest and most underprivileged ethnic minority [5]. Due to a lack of official documentation and fear of stigmatisation, the true size of the Roma population is unknown today, but it is estimated that between 10 and 12 million Roma live in Europe [6], with the majority in the central and eastern regions of the continent, accounting for more than 5% of the total population [7]. In 2022, the Roma represented 2.2% of the total population of Hungary, with 209,909 self-reported Roma people [5]. Across Europe, the Roma minority has faced decades of discrimination, with a lack of privilege linked to lower levels of education compared to the general population, higher unemployment rates, poverty and poorer health status [8–10]. Roma people usually live in so called ‘underprivileged’ areas in Hungary. An underprivileged area is defined as one with a low employment rate, high rate of unqualified labour, low quality of life and living standards, poor access to public services, low economic performance and lack of basic infrastructure [11]. Access to health care for the Roma population is limited and, as a probable consequence, most Roma cardiovascular patients have poorer cardiometabolic status at the time of initiation of care than the majority population [12]. The prevalence of certain diseases, such as communicable diseases, CVD, bronchial asthma and diabetes, is also higher among the Roma population [13].

Previous studies have already indicated that the Roma population is more exposed to certain risk factors for CVD compared to the general population, including heavy smoking, obesity, diabetes, insulin resistance, hypertension, metabolic syndrome, dyslipidaemia and physical inactivity [14, 15].

The Roma ethnicity in Hungary is mainly concentrated in remote settlements, most of which are underprivileged. We wanted to explore in detail the factors that contribute to their known poorer cardiovascular status.

Thus, this study aimed to investigate the health status and cardiovascular risk factors of the population aged 18 years and over undergoing cardiovascular screening in underprivileged settlements in Hungary, with a special focus on the differences between the Roma and non-Roma populations. In addition, it compared information on the cardiovascular risk factors (such as obesity, hypertension and diabetes) of people living in underprivileged municipalities with similar data from the general Hungarian population.

## Methods

Several central initiatives have been undertaken in Hungary to reduce morbidity and mortality related to CVD, including Government Resolution No. 1057/2021 (II.19) on a development program necessary to reduce economic inequalities between certain areas of Hungary, as well as a government resolution on the extension of the long-term program ‘Rising Settlements’, primarily tasked with providing cardiovascular screening for residents in the less developed regions of Hungary. The realisation of this goal was facilitated by the governmental screening program called ‘Taking the screening tests close to the people’.

The program was implemented in collaboration with the National Public Health Centre, the Maltese Charity Service and the Gottsegen National Cardiovascular Center (GOKVI) in Budapest, which also provided the health specialists – cardiologists and other health specialists – at the screening sites. In each place, the screening team was led by a cardiology specialist and included a cardiology resident and staff from the nursing and administration service.

During the 4 years of the program, a total of 197 underprivileged settlements were visited by the cardiovascular screening and counselling buses free of charge. More than 10,000 applicants over the age of 18 requested this service.

This article reviews the ‘Taking the screening tests close to the people’ program data collected in 2021 and 2022 and compares them with relevant national data. In these 2 years, screenings were carried out in a total of 115 underprivileged Hungarian settlements, mostly in border areas. At the time of the screenings, 40% of the participating settlements had a vacant general practitioner’s (GP) practice, that is, the practice was being permanently covered by a doctor from another practice with limited local presence or the doctor’s office was located in a neighbouring or distant settlement. For the inhabitants of underprivileged settlements, access to health care is limited due in part to the abovementioned reason and partly because of transportation difficulties or other unfavourable conditions.

Patients visited the screening points on a voluntary basis, having been informed in advance by the local municipality and the GP service (posters, online advertisements and promotional films). In the first round, a 44-item questionnaire was administered to all applicants aged 18 and over who gave written consent to participate in the screening and the study. The questionnaire was developed for this study (see in the appendix). Most of the questions regarding health status were extracted from the validated questionnaire used by the European Health Interview Surveys executed in Hungary. The questionnaire itself contained questions on several topics validated for the Hungarian language, such as physical activity or smoking [16]. Key demographic data (gender, age, ethnicity, educational level and family status), self-reported medical history (current and past medical conditions) and lifestyle information (dietary habits and smoking) were recorded. Two questions clarifying ethnicity were asked, one on self-reported primary ethnicity (‘What ethnicity do you feel to belong to?’) and the other on self-reported secondary ethnicity (‘Do you identify as any other ethnicity?’). Based on the question about educational attainment (‘What is your highest level of education?’), three categories were distinguished: primary education (8 grades or less), secondary education and tertiary education (university or college). To the question about physical activity (‘How much time did you spend in the past week doing the following activities: swimming, jogging, aerobics, football, gym, training, etc.?’), there were four possible answers: none, less than 1 h, 1–3 h and more than 3 h. Here, a minimum of 3 h of exercise per week and less than 3 h of exercise per week were examined. Smoking was investigated with the question ‘Do you currently smoke?’, to which there were five possible answer options: yes, daily; yes, occasionally; I have never smoked; no, I stopped less than 1 year ago; and no, I stopped more than 1 year ago. Those reporting daily smoking were considered active smokers and others were categorised as non-smokers. In the case of anamnestic diseases (‘Do you have any of the diseases listed below?’) with the responses yes, no and I do not know, the prevalence of hypertension and type 2 diabetes were examined based on self-declaration. The questionnaire was followed by a general health assessment, which included body weight, body height, blood pressure, blood sugar and ankle-brachial index measurements.

Blood pressure was measured after 5–10 min at rest using a certified automated blood pressure monitor (Omron M3500; Kyoto, Japan) with an appropriate cuff size, with readings taken on both arms. Blood pressure was considered elevated if the systolic value exceeded 140 mmHg and/or the diastolic value exceeded 90 mmHg. Blood glucose levels were measured from fingerstick blood (Wellmed Easy Touch ET-GC; San Antonio, TX, USA) and whether each measurement was taken on an empty stomach was recorded. Blood glucose was considered high if the fasting value was above 5.0 mmol/l or the random blood glucose was above 11.1 mmol/l at any time. Atrial fibrillation was screened by electrocardiogram (ECG) and physical examination. The ankle-arm index was measured using a MESI ABPI MD device (Ljubljana, Slovenia) with an appropriate cuff size. At the last stage of the screening, after the 12-lead ECG recording (EDAN SE-1200 ECG machine; Shenzhen, China), participants were examined by a cardiologist. Echocardiography (LOGIQ V2 ultrasound machine; General Electric, Boston, MA, USA) was performed on site, and if professionally indicated, medication was modified or patients were referred to further specialist care (e.g., internal medicine, diabetology, pulmonology, cardiology, angiology, emergency department). The devices used for the measurements were all certified.

### Statistical methods

Normality was tested with the Shapiro–Wilk test for continuous variables. Since most of the variables did not have a normal distribution, the two independent groups’ data were analysed with non-parametric Mann–Whitney U tests. Continuous data were presented as medians and interquartile ranges. Categorical data were analysed with Pearson’s chi-square test and presented as strata-specific case counts and proportions. Finally, three individual multiple logistic regression models were executed with enter method to identify the factors that might contribute to an elevated body mass index (BMI), diabetes or hypertension in separate confounder-adjusted models. The explanatory variables were the same in all three models, with one exception; in one model where the elevated BMI was the outcome variable, BMI (measured on a continuous scale) was not included as an explanatory variable, but otherwise the following explanatory variables were included: ethnicity, age, gender, educational attainment, smoking status, physical activity. Adjusted odds ratios (AOR) with their corresponding 95% confidence intervals were calculated for the multiple models, for other statistics *p* < 0.05 was considered significant.

## Results

### Descriptive statistics

Data from 6211 participants living in underprivileged settlements were analysed. A total of 16.1% (859) of the participants identified themselves as Roma (200 (23.3%) men and 659 (76.7%) women). The non-Roma population comprised 5352 participants, of whom 1364 (25.5%) were men and 3988 (74.5%) were women (*p* < 0.001). There was a significant difference in the median age between the non-Roma and Roma populations (59.0 years (48.0–67.9) compared to only 49.3 years (38.4–58.7); *p* < 0.001).

Significant differences were observed regarding educational level between the non-Roma and Roma populations (*p* < 0.001). In the non-Roma population, 29.4% (1569) of the participants had primary education as the highest educational attainment; in the Roma population, the figure was 77.2% (660). Of the non-Roma population, 53.9% (2874) and of the Roma population, 21.4% (183) completed secondary school. While 16.7% (889) of the non-Roma population had a college or university degree, only 1.4% (12) of the Roma population did.

There was a significant difference in physical activity between the two investigated populations (*p* < 0.001). Of the non-Roma population, 8.8% (468) and of the Roma population, 7.5% (64) exercised at least 3 h per week. The proportion of those who exercised less than 3 h per week was 91.2% (4849) in the non-Roma population and 92.5% (788) in the Roma population.

In the non-Roma population, 71.7% (3512) had a BMI above 25 kg/m^2^, while 72.4% (609) of the Roma population were overweight or obese (*p* = 0.709). There was a significant difference between the two populations in the median BMI (*p* < 0.001), which was 28.0 kg/m^2^ (24.6–31.9) in the non-Roma population and 28.8 kg/m^2^ (24.5–33.0) in the Roma population.

There was a significant difference in active smoking between the non-Roma and Roma populations (*p* < 0.001): 28.7% (1531) of the non-Roma population smoked regularly (that is, every day), compared to 60.3% (516) of the Roma population.

Significant differences were observed regarding hypertension between the non-Roma and Roma populations (*p* < 0.001). The prevalence of the hypertension was 54.9% (2824) in the non-Roma population and 49.8% (412) in the Roma population. Significant differences were also observed regarding diabetes between the Roma and non-Roma populations (*p* < 0.001). The prevalence of diabetes was 12.2% (619) in the non-Roma population and 11.5% (95) in the Roma population (Tables 1 and 2).

**Table 1:** Descriptive results of the demographic features, lifestyle and some current illnesses of the study population

		Roma	Non-Roma	*p*-value
Median age (years) – interquartile range		49.3 (38.4–58.7)	59.0 (48.0–67.9)	*p* < 0.001
Education	Primary	77.2% (660)	29.4% (1569)	*p* < 0.001
	Secondary	21.4% (183)	53.9% (2874)	
	Tertiary	1.4% (12)	16.7% (889)	
Exercise	> 3 h/ week	7.5% (64)	8.8% (468)	*p* < 0.001
	< 3 h/ week	92.5% (788)	91% (4849)	
BMI (kg/m2)	Above 25	72.4% (609)	71.7% (3512)	*p* = 0.709
	Belove 25	27.6% (232)	28.3% (1386)	
Median BMI (kg/m2) – interquartile range		28.0 (25.6–31.9)	28.8 (24.5–33.0)	*p* < 0.001
Smoking	Every day	60.3% (516)	28.7% (1531)	*p* < 0.001
	Not every day	39.7% (340)	71.3% (3811)	
Hypertension	Yes	49.8% (412)	54.9% (2824)	*p* < 0.001
	No	50.2% (416)	45.1% (2316)	
Diabetes (Type2)	Yes	11.5% (95)	12.2% (619)	*p* < 0.001
	No	88.5% (732)	87.8% (4454)	

The sample numbers are in the brackets

### Multiple regression results

#### BMI above 25 kg/m^2^

Members of the Roma population (AOR = 1.47 [1.22–1.77]) had a significantly higher prevalence of overweight and obesity (BMI > 24.9 kg/m^2^) compared to members of the non-Roma population. This condition also became more prevalent with age, with the odds of overweight and obesity increasing by 1% for each year of life (AOR = 1.01 [1.01–1.02]). Women were significantly less likely to have a BMI over 24.9 kg/m^2^ (AOR = 0.71 [0.62–0.82]) compared to men. Those with tertiary education were 30% less likely to have a high BMI compared to those with primary education (AOR = 0.70 [0.57–0.84]). There was no significant association between secondary education and the development of overweight or obesity (AOR = 1.07 [0.93–1.23]), but it did appear to be more common among those with secondary education compared to those with primary education. Regular smokers had a significantly lower prevalence of overweight and obesity (AOR = 0.53 [0.46–0.60]) than non-smokers. Exercising more than 3 h per week reduced the odds of overweight and obesity (AOR = 0.72 [0.59–0.88]) compared to exercising less than 3 h per week.

#### Diabetes

There was no significant association between ethnicity and the likelihood of developing diabetes, with approximately the same odds of developing the disease in both the Roma (AOR = 0.99 [0.75–1.31]) and non-Roma populations. Older age was a risk factor for diabetes, with older patients having a significantly higher odds of developing the disease and the odds of the disease increasing by 5% for each year of life (AOR = 1.05 [1.04–1.06]). No significant association was found between gender and the onset of diabetes, but women were less likely to develop the disease (AOR = 0.90 [0.74–1.09]). Both secondary (AOR = 0.64 [0.53–0.77]) and tertiary education (AOR = 0.50 [0.36–0.69]) were identified as protective factors for the onset of diabetes compared to primary education. The prevalence of the disease increased with higher BMI, with each 1 kg/m^2^ increase in BMI increasing the odds of diabetes by 10% (AOR = 1.10 [1.08–1.11]). No significant association was found between smoking and the development of diabetes, but the prevalence of the disease appeared to be higher among regular smokers (AOR = 1.16 [0.94–1.42]). There was no significant association between physical activity and the development of diabetes, with approximately equal odds of developing the disease between those who exercised at least 3 h per week (AOR = 0.99 [0.71–1.37]) and those who exercised 3 h or less per week.

#### Hypertension

In our study, no significant association between ethnicity and prevalence of hypertension was found, but members of the Roma population seemed to have higher odds of developing the disease (AOR = 1.19 [0.97–1.45]) than the non-Roma population. Our data showed that the prevalence of hypertension increased significantly with age, with the odds of the disease increasing by 8% for each year of life (AOR = 1.08 [1.07–1.09]). Women were 19% more likely to have hypertension (AOR = 1.19 [1.03–1.38]) than men. The disease was 24% less likely to occur in those with secondary education (AOR = 0.76 [0.65–0.88]) and 46% less likely to occur in those with tertiary education (AOR = 0.54 [0.44–0.67]) compared to those with up to 8 years of primary education. The prevalence of hypertension increased with increasing BMI, with an 11% increase in the odds of hypertension with each 1 kg/m^2^ increase in BMI (AOR = 1.11 [1.10–1.13]). There was no significant association between smoking and the incidence of hypertension (AOR = 1.00 [0.87–1.15]). There was also no significant association between physical activity and the development of hypertension, but a minimum of 3 h of physical activity per week appeared to reduce the likelihood of developing the disease (AOR = 0.81 [0.65-1.00]).

**Table 2:** Factors associated with obesity, diabetes and hypertension based on the multiple and multivariate logistic regression results

	BMI > 24.9			Diabetes (Type 2)			Hypertension		
	AOR	95% Confidence Interval		AOR	95% Confidence Interval		AOR	95% Confidence Interval	
Roma vs. non-Roma	**1.47**	**1.22**	**1.77**	0.99	0.75	1.31	1.19	0.97	1.45
Age - Years	**1.01**	**1.01**	**1.02**	**1.05**	**1.04**	**1.06**	**1.08**	**1.07**	**1.09**
Sex - Women vs. Men	**1.71**	**0.62**	**0.82**	0.90	0.74	1.09	**1.19**	**1.03**	**1.38**
Education - Secondary vs. Primary	1.07	0.93	1.23	**0.64**	**0.53**	**0.77**	**0.76**	**0.65**	**0.88**
Education - Tertiary vs. Primary	**0.70**	**0.57**	**0.84**	**0.50**	**0.36**	**0.69**	**0.54**	**0.44**	**0.67**
Smoking - Yes vs. No	**0.53**	**0.46**	**0.60**	1.16	0.94	1.42	1.00	0.87	1.15
Physical activity - Minimum 3 h per week vs. Maximum 3 h per week	**0.72**	**0.59**	**0.88**	0.99	0.71	1.37	0.81	0.65	1.00
BMI				**1.10**	**1.08**	**1.11**	**1.11**	**1.10**	**1.13**

Significant results are marked in Bold

## Discussion

### Characteristics of the underprivileged Roma population

Based on our findings, members of the Roma population were significantly less likely to have completed secondary or tertiary education compared to the non-Roma population. Based on a study published in 2018, those with higher education have a higher prevalence of health literacy compared to those with only primary education. In addition, a lower socioeconomic status is associated with lower life expectancy and age at onset of certain morbidities. Individuals who have completed tertiary education are more likely to have better health status than those with lower levels of education [17].

Based on logistic regression results adjusted for confounders, we came to the following conclusions. Higher education was identified as a protective factor for overweight and obesity compared to primary education. For hypertension and diabetes, both tertiary and secondary education were protective factors compared to primary education. These diseases were significantly less prevalent among those with more than 8 years of primary education.

Smoking is an important determinant of health behaviour: 18.4% of the population smokes daily in Europe [18], while in Hungary the prevalence is 24.5% [19]. While the prevalence in the Hungarian non-Roma population of the study was found to almost equal the national average (28.7%), the prevalence in the Roma population in underprivileged regions was much higher, with 60.3% of the population smoking daily. Another indicator of health behaviour is the frequency of physical activity, with almost a third of the Hungarian population completing at least 150 min of physical activity per week [20], which is much lower than the European average, while positive trends can be observed in Central European countries undergoing similar political and social changes as in Hungary, where 57–72% of the population regularly do some form of physical activity [21]. In contrast, even lower rates were found in the underprivileged Hungarian population, with 91.2% of the non-Roma population reporting less than 3 h of physical activity per week, compared to 92.5% of the Roma population.

Lifestyle plays a major role in the development of higher BMI, which is a trait determined partly by genetics and partly by lifestyle factors. In Europe, the prevalence of overweight and obesity is 52.7% [22], while in Hungary 62% of the population has a BMI above 25 kg/m^2^ [23]. The BMI values of people living in underprivileged settlements were almost 10% higher than those of the average Hungarian population, with 71.7% of the non-Roma population having a BMI above 25 kg/m^2^, while 72.4% of the Roma population were overweight or obese. The prevalence of overweight and obesity in the underprivileged Hungarian population is well above the prevalence rates measured worldwide. Members of the Roma population had a significantly higher median BMI (28.8 kg/m^2^) than the non-Roma population (28.0 kg/m^2^).

Based on the logistic regression results, a BMI above 25 kg/m^2^ was significantly more frequent in the Roma population than in the non-Roma population. In a survey conducted in 2023 in Hungary, Romania and Slovakia, the Roma population had a similar significantly higher mean BMI compared to the non-Roma population [12]. The prevalence of overweight and obesity increased with age. In our study, we found that the odds of developing the condition increased by 1% per year for overweight and obesity. A BMI over 25 kg/m^2^ and hypertension were significantly more common in women than in men in our study population.

The international (31%) and Hungarian (35%) data are almost identical regarding the prevalence of hypertension [24, 25]. In contrast, the prevalence of the disease was much higher among the Hungarian population living in underprivileged settlements, which may consistent with their poorer health status. Hypertension was significantly more common in the non-Roma population (54.9%) compared to the Roma population (49.8%).

For diabetes, similar results were found, with a prevalence of 9.3% worldwide and 7.51% in Hungary [26]. The prevalence of the disease was also significantly higher in the study population compared to the Hungarian average, with diabetes being significantly more prevalent in the non-Roma population (12.2%) compared to the Roma population (11.5%).

Based on the logistic regression results, the prevalence of both hypertension and diabetes increased with increasing BMI. Several studies have demonstrated that overweight and obesity are significant risk factors for both hypertension and diabetes [24, 26]. For hypertension and diabetes, no significant association was found between these diseases and ethnicity. In addition, the prevalence of hypertension and diabetes increased with age. In our study, we found that the odds of developing the condition increased with each year of life by 8% for hypertension and 5% for diabetes. Other international studies have also shown that the prevalence of hypertension and diabetes increases with age [24, 26].

### Limitations

The selection of participants was done consecutively, with applicants voluntarily presenting themselves at the screening points. As these screening examinations were organised with the aim of upgrading their health awareness in these areas, the sample cannot be considered representative for Roma people or the entire Hungarian population. Furthermore, women – generally, regarding the use of healthcare services – participated in the examination at a significantly higher rate than men. The completion of the questionnaires was based on self-reporting, including ethnicity and previous or current illnesses. However, healthcare professionals sought to overcome this limiting factor by providing thorough explanations of the questions, and participants could answer questions related to sensitive topics (such as ethnicity and smoking) without the presence of the interviewer.

## Conclusions

The prevalence of certain chronic diseases and cardiovascular risk factors is much higher in the Hungarian population than in other European countries. In terms of both health status and lifestyle risks, people living in underprivileged settlements are worse off than the Hungarian national average: members of the Roma population, although much younger in this study, showed significantly worse characteristics than the non-Roma population in terms of lifestyle risks and certain cardiovascular risk conditions.

In the underprivileged population, a high prevalence of overweight and obesity was observed, being 71–72% among both the Roma and non-Roma populations, which is even 10% higher than the very unfavourable Hungarian average. A lack of physical activity (91–92% among both the Roma and non-Roma populations) and an extremely high smoking rate, comprising 60% of the underprivileged Roma population, were also observed. Both type 2 diabetes and hypertension were more common in people living in underprivileged settlements than in the average Hungarian population (diabetes: 11–12% vs. 7.51%; hypertension: 50–55% vs. 35%).

Underprivileged populations, including the Roma population, had a much higher prevalence of CVD risk factors compared to the majority population. Given the high prevalence of cardiovascular risk factors among Roma populations living in deprived settlements, complex interventions based on targeted cooperation between healthcare and social professionals are needed.

### Electronic supplementary material

Below is the link to the electronic supplementary material.

Supplementary Material 1

## Data Availability

The dataset used and/or analysed during the current study are available from the corresponding author on reasonable request.

## Abbreviations

AOR: Adjusted odds ratio
BMI: Body-mass index
CVD: Cardiovascular diseases
ECG: Electrocardiogram
GOKVI: Gottsegen National Cardiovascular Center
GP: General Practice
