# Supplementary material for: Cardiovascular risk factors among Roma and non-Roma populations in underprivileged settlements
Source: BMC Prim Care. 2024 Aug 8;25:289. doi: 10.1186/s12875-024-02555-6 (PMC11312200; doi:10.1186/s12875-024-02555-6)
Supplement: Supplementary file 1 — Supplementary Material 1 [file 12875_2024_2555_MOESM1_ESM.pdf]

TAKING THE SCREENING TESTS CLOSE TO THE PEOPLE

GOKVI

LOCATION, DATE: ....

---

1. **Patient ID:** ....
2. **Birth date** (year. month. day.): ....
3. **Sex:** ....
4. **Zip code:** ....

5. **What ethnicity do you feel to belong to?**

1 – Hungarian
2 – Bulgarian
3 – Roma
4 – Greek
5 – Croatian
6 – Polish
7 – German
8 – Armenian
9 – Romanian
10 – Ruthenian
11 – Serbian
12 – Slovak
13 – Slovenian
14 – Ukrainian
15 – Arabic
16 – Chinese
17 – Russian
18 – Vietnamese
19 – Other:

6. **Do you identify as any other ethnicity?**

0 – Does not belong to any other ethnicity
1 – Hungarian
2 – Bulgarian
3 – Roma
4 – Greek
5 – Croatian
6 – Polish
7 – German
8 – Armenian
9 – Romanian
10 – Ruthenian
11 – Serbian
12 – Slovak
13 – Slovenian
14 – Ukrainian
15 – Arabic
16 – Chinese
17 – Russian
18 – Vietnamese
19 – Other:

7. **What is your highest level of education?**

1 - 8 grades or less
2 - secondary education
3 - tertiary education (university, collage)

8. **What is your marital status?**

1 - single
2 - married/ partnered
3 - divorced
4 - widow

---

9. **Do you currently smoke?**

1 - yes, daily
2 - yes, occasionally
3 - I have never smoked
4 - no, I stopped less than a year ago
5- no, I stopped more than a year ago

10. **How many cigarettes do you smoke on average per day?**

1 - 0
2 - from 1 to 9 cigarettes
3 - from 10 to 19 cigarettes
4 - over 20 cigarettes

11. What is the extent of physical activity during your work? Mark only one answer!

1 - I do not work (e.g. retired, unemployed, stay-at-home mom, etc.)
2 - I sit most of my work time (e.g. office worker)
3 - I stand or walk most of my work time. However, my work does not require a lot of physical effort (e.g. shop assistant, hairdresser, security guard, childcare, etc.)
4 - My job requires a lot of physical effort, including lifting heavy objects and using tools (e.g. plumber, electrician, carpenter, cleaner, nurse, gardener, postman, etc.)
5- My job requires a lot of physical effort, including lifting very heavy objects (e.g. construction worker, dustman, etc.)

12. How much time did you spend in the past week doing the following activities: swimming, jogging, aerobics, football, tennis, gym training, etc.?

1 - none
2 - less than 1 hour
3 - 1-3 hours
4 - more than 3 hours

13. How much time did you spend in the past week doing the following activities: cycling, including to and from work and for leisure?

1 - none
2 - less than 1 hour
3 - 1-3 hours
4 - more than 3 hours

14. How much time did you spend in the past week doing the following activities: walking, including to and from work, for shopping and for leisure?

1 - none
2 - less than 1 hour
3 - 1-3 hours
4 - more than 3 hours

15. In the past week, how much time did you spend on: housework/childcare?

1 - none
2 - less than 1 hour
3 - 1-3 hours
4 - more than 3 hours

16. In the past week, how much time did you spend on: gardening or „do it yourself" activities?

1 - none
2 - less than 1 hour
3 - 1-3 hours
4 - more than 3 hours

17. How much time did you spend sitting last week (including during work, leisure activities, transport)?

1 - less than 3 hours
2 - 3-5 hours
3 - 5-8 hours
4 - more than 8 hours

18. How often do you eat fruits and vegetables?

1 - every day
2 - not every day

19. How often do you eat sweets (e.g. chocolate, biscuits, cakes, ice cream, etc.)?

1 - never
2 - up to 3 times a week
3 - 4 or more times a week
4 - occasionally

20. How often do you eat salty snacks (e.g. peanuts, chips, etc.)?

1 - never
2 - up to 3 times a week
3 - 4 or more times a week

4 - occasionally

21. How often do you drink sugary drinks (e.g. Coke, Fanta, Sprite, Iced tea, etc.)?

1 - never
2 - up to 3 times a week
3 - 4 or more times a week
4 - occasionally

22. How often do you drink energy drinks (e.g. Red Bull, Hell, Burn, etc.)?

1 - never
2 - up to 3 times a week
3 - 4 or more times a week
4 - occasionally

23. How often do you drink alcohol?

1 - never
2 - monthly or less
3 - twice or four times a month
4 - twice or three times a week
5- four or more times a week

24. Have you ever felt the need to cut back on the amount of alcohol you used to drink?

1 - yes
2 - no
3 - I cannot answer
4 - I do not want to answer

25. Looking back over the last 12 months, how do you identify your health status?

1 - very good
2 - good
3 - average
4 - poor
5 - very poor
6 - I cannot answer
7 - I do not want to answer

Do you have any of the diseases listed below?

**26. Do you have hypertension?**	1 – yes	2 – no	3 – I don't know
**27. Do you have any chronic pulmonary diseases?**	1 – yes	2 – no	3 – I don't know
**28. Do you have any coronary diseases?**	1 – yes	2 – no	3 – I don't know
**29. Do you have any cerebrovascular diseases?**	1 – yes	2 – no	3 – I don't know
**30. Do you have peripheral artery disease?**	1 – yes	2 – no	3 – I don't know
**31. Do you have Type 2 diabetes?**	1 – yes	2 – no	3 – I don't know
**32. Do you have Type 1 diabetes?**	1 – yes	2 – no	3 – I don't know
**33. Do you have any chronic kidney diseases?**	1 – yes	2 – no	3 – I don't know
**34. Do you have any mental disorders (depression, anxiety, etc.)?**	1 – yes	2 – no	3 – I don't know
**35. Had anyone in your family (siblings, parents, grandparents) under the age of 50 a heart attack or stroke?**	1 – yes	2 – no	3 – I don't know
**36. Do you have any other diseases?**	1 - yes	2 – no	3 – I don't know

Other diseases:

37. . Are you being treated for a chronic disease?

1 - yes
2 - no

38. Who is mainly responsible for your treatment?

1 - GP
2 - medical specialist
3 - both of them

39. Does your disease make it difficult for you to go about your daily life?

1 - yes
2 - no

40. Do you have any help with your disease in everyday life?

1 - yes, my family members help
2 - yes, in the form of home care
3 - yes, neighbours and friends help
4 - I have no help

41. Do you have any problems getting to the place of health care?

1 - no
2 - yes, because of financial problems
3 - yes, because of reduced mobility
4 - yes, because the medical institution is far away
5 - yes, because I do not have enough time
6 - yes, because I cannot make an appointment
7 - yes, because there is no such care in the area (within 60 km)
8 - yes, because I have no help

42. What information was given to you about your disease during your health care?

1 - I have been fully informed
2 - I also received full information and patient education
3 - I was partially informed, but it would be good to know more about it
4 - I do not know what my disease is, I have not been informed

43. Apart from health care specialists, from whom or where did you get information about your disease?

1 - family, friends
2 - newspapers
3 - television, radio
4 - internet
5 - I have not checked
6 - I have not found any information about it

44. Do you follow the treatment plan?

1 - yes
2 - partly
3 - no, because I disagree with the treatment
4 - no, because I cannot afford the medicines
5 - no, because there was a side effect
6 - no, because I don't know how to use it
7 - no, because I do not trust its effectiveness
8 - no, because I am afraid of side effects

Measured results:

Body weight		kg	
Body height		cm	
Waist circumference		cm	
Blood pressure left arm, systolic		mmHg	
Blood pressure left arm, diastolic		mmHg	
Ankle-brachial index, right		mmHg	
Ankle-brachial index, left		mmHg	
Total cholesterol		mmol/l	
Are you treated for high cholesterol?	*yes*	*no*	*not relevant*
Blood glucose		mmol/l	
Was it recorded on an empty stomach?	*yes*	*no*	
Are you treated for high blood glucose?	*yes*	*no*	*not relevant*
